# The quality of mental health care for people with bipolar disorders in the Italian mental health system: the QUADIM project

**DOI:** 10.1186/s12888-023-04921-7

**Published:** 2023-06-13

**Authors:** Barbara D’Avanzo, Angelo Barbato, Matteo Monzio Compagnoni, Giulia Caggiu, Liliana Allevi, Flavia Carle, Teresa Di Fiandra, Lucia Ferrara, Andrea Gaddini, Michele Sanza, Alessio Saponaro, Salvatore Scondotto, Valeria D Tozzi, Cristina Giordani, Giovanni Corrao, Antonio Lora

**Affiliations:** 1 Department of Health Policy, Istituto di Ricerche Farmacologiche Mario Negri IRCCS, Milano, Italy; 2 National Centre for Healthcare Research and Pharmacoepidemiology, University of Milano-Bicocca, Milan, Italy; 3 Unit of Biostatistics, Epidemiology and Public Health, Department of Statistics and Quantitative Methods, University of Milano-Bicocca, Street Bicocca degli Arcimboldi, 8, Building U7, Milan, 20126 Italy; 4 Center of Epidemiology and Biostatistics, Polytechnic University of Marche, Ancona, Italy; 5 National Institute of Health, Rome, Italy; 6 Centre of Research on Health and Social Care Management, SDA Bocconi School of Management (Bocconi University, Milan, Italy; 7 Agency for Public Health, Lazio Region, Rome, Italy; 8 Department of Mental Health and Substance Abuse, Local Health Trust of Romagna, Cesena, Italy; 9 General Directorate of Health and Social Policies, Emilia-Romagna Region, Bologna, Italy; 10 Department of Health Services and Epidemiological Observatory, Regional Health Authority, Sicily Region, Palermo, Italy; 11 Department of Health Planning, Italian Health Ministry, Rome, Italy; 12 Department of Mental Health and Addiction Services, ASST Lecco, Lecco, Italy

**Keywords:** Healthcare utilization databases, Bipolar disorders, Quality of mental health care, Treatment gap, Clinical pathways, Mental health care, Real-world, Healthcare research, Public health, Healthcare services

## Abstract

**Background:**

The assessment of the quality of care pathways delivered to people with severe mental disorders in a community-based system remains uncommon, especially using healthcare utilization databases. The aim of the study was to evaluate the quality of care provided to people with bipolar disorders taken-in-care by mental health services of four Italian areas (Lombardy, Emilia-Romagna, Lazio, province of Palermo).

**Methods:**

Thirty-six quality indicators were implemented to assess quality of mental health care for patients with bipolar disorders, according to three dimensions (accessibility and appropriateness, continuity, and safety). Data were retrieved from healthcare utilization (HCU) databases, which contain data on mental health treatments, hospital admissions, outpatient interventions, laboratory tests and drug prescriptions.

**Results:**

29,242 prevalent and 752 incident cases taken-in-care by regional mental health services with a diagnosis of bipolar disorder in 2015 were identified. Age-standardized treated prevalence rate was 16.2 (per 10,000 adult residents) and treated incidence rate 1.3. In the year of evaluation, 97% of prevalent cases had ≥ 1 outpatient/day-care contacts and 88% had ≥ 1 psychiatric visits. The median of outpatient/day-care contacts was 9.3 interventions per-year. Psychoeducation was provided to 3.5% of patients and psychotherapy to 11.5%, with low intensity. 63% prevalent cases were treated with antipsychotics, 71.5% with mood stabilizers, 46.6% with antidepressants. Appropriate laboratory tests were conducted in less than one-third of prevalent patients with a prescription of antipsychotics; three quarters of those with a prescription of lithium. Lower proportions were observed for incident patients. In prevalent patients, the Standardized Mortality Ratio was 1.35 (95% CI: 1.26–1.44): 1.18 (1.07–1.29) in females, 1.60 (1.45–1.77) in males. Heterogeneity across areas was considerable in both cohorts.

**Conclusions:**

We found a meaningful treatment gap in bipolar disorders in Italian mental health services, suggesting that the fact they are entirely community-based does not assure sufficient coverage by itself. Continuity of contacts was sufficient, but intensity of care was low, suggesting the risk of suboptimal treatment and low effectiveness. Care pathways were monitored and evaluated using administrative healthcare databases, adding evidence that such data may contribute to assess the quality of clinical pathways in mental health.

**Supplementary Information:**

The online version contains supplementary material available at 10.1186/s12888-023-04921-7.

## Background

Despite evidence of some impact of care on mental health, quality of care improvement in this field have been limited, or even worsened in some cases [1, 2] in comparison to other medical conditions [3]. This gap is observed in general and specific conditions as well. Data from the 2017 Global Burden of Disease Study showed an age-standardized rate of disability-adjusted life years (DALYs) of 176/100,000 individuals in Western Europe. Bipolar disorder is a relevant cause of disability worldwide throughout adult life, especially in people aged 20–44 [4], and is associated with high rates of premature mortality mainly due to medical comorbidities, such as cardiovascular diseases [5].

One-year prevalence was estimated 0.6% in males and 0.8% in females in Western Europe [6]. A prevalence around 2% was found in North American studies [7], whereas for treated prevalence a North American study showed a 12-month rate of 0.5% [8], indicating a treatment gap of about 75% compared to the true prevalence in the same region. Lifetime treatment rates of bipolar disorders in high income countries were estimated at 50% or less than true rates [7].

The recurrence rate for individuals recovered from first manic episode estimated around 46% within two years highlights the limitations of current available treatments [9, 10]. Moreover, care offered to bipolar disorders is often suboptimal, and an implementation gap exists in psychosocial [11] and pharmacological [12] treatments.

In the last decades, thanks to the increasing availability of real-world data [13–15] the knowledge of patterns of care could be retrieved more rapidly, enabling comparisons [16]. This is especially important for community-oriented care, since community care is managed at regional and local level, which can introduce difficulties in equity in service delivery and integration and comparability of information. This is the case of Italy, where in 1978 a reform law (e.g., “Law Number 180”) blocked all the new admissions to public psychiatric hospitals, encouraging the implementation of a widespread and structured network of community mental health facilities. Indeed, in Italy a community model was implemented earlier than in most countries and during the last 40 years efforts were made to move away more thoroughly from the institutional-based approach [17], with a progressive consolidation of a community-based system of mental health care. In Italy, the National Health System (NHS) is organized in public Local Health Authorities, each one including one Department of Mental Health (DMH), which provides comprehensive mental healthcare for the target population. Each DMH manages a local network of community services (such as Community-Mental-Health Centres (CMHCs), General Hospital Psychiatric Wards (GHPWs), Day-Care Centres (DCs), and Community-Residential Facilities (CRFs)) on a unitary basis, and these must provide at least the minimum set of services required by law [18]. Private healthcare providers deliver day-care and residential care in conjunction with public DMHs.

Given these premises, the Italian Ministry of Health funded the QUADIM Project, a multi-regional investigation based on real-world data retrieved from administrative databases, to monitor and evaluate the mental healthcare for people with schizophrenic, bipolar, depressive and personality disorders [19, 20]. Here we present the findings related to bipolar disorders.

## Methods

### Aim

The aim of the current study is to evaluate the quality of mental healthcare delivered to patients with bipolar disorders taken-in-care by Italian public services of mental health, using healthcare utilization databases.

### Setting / data sources

The QUADIM project retrieved data retrospectively from the computerized healthcare utilization (HCU) databases of four Italian regions, i.e., Lombardy, Emilia-Romagna, Lazio, and Sicily (province of Palermo); available for the 2013–2016 time interval at the beginning of the project.

The HCU data, used to locally manage information on healthcare provided, include an array of information on residents who receive care: discharges from public or private hospitals, drug prescriptions in any hospital, residential or outpatient setting, specialist visits and diagnostic exams reimbursable by the NHS. Furthermore, a national specific information system of psychiatric care (MHIS) collects information provided by the DMHs and private care facilities accredited by the NHS (except for office-based private practice and primary care). As part of the MHIS, socio-demographic information, ICD-10 or ICD-9-CM diagnoses, day-care attendances, admissions to GHPWs and CRFs for patients receiving mental health care are recorded. The codes used to draw records and fields from databases are reported in Supplementary Tables S1 and S2. As a unique identification code, anonymized for privacy issues, is used for all databases within each region, it was possible to link HCU databases through a record-linkage procedure, allowing to describe the complete care pathway of each user. Details of HCU databases use in mental health were reported elsewhere [19–22]. Although differences in the databases across regions were limited, a between-region data harmonization was performed allowing the implementation of consistent and comparable data extraction processes (e.g., information of datasets and variables was uniformly encoded by using the same names, values and formats, etc.). Based on a detailed protocol detailing data harmonization and extraction processes, regional anonymized data were extracted and processed locally by using common Statistical Analysis System (SAS) programs developed by two of the authors (Monzio Compagnoni and Caggiu).

### Cohort selection

The target population included all beneficiaries of the NHS resident in Lombardy, Emilia-Romagna, Lazio, and province of Palermo aged 18–65 years. According to the Italian Institute of Statistics, this population amounted to 13.5 million people [http://demo.istat.it/index.html]. Among individuals with a diagnosis of bipolar disorder we identified those who had at least one contact with a DMH from January to December 2015, labelled as *treated prevalent cases*. The date of their first contact with DMHs during the considered period was recorded as index date. The cohort of newly taken-in-care patients (labelled *treated incident cases*) included individuals aged 18–40 years at their first contact with DMHs, excluding those who (i) received a diagnosis of any mental disorder at any time prior to the index date, (ii) experienced any hospital admission to a GHPW, or (iii) received at least two prescriptions of antipsychotic drugs or mood stabilizers in the two years before the index date.

Members of both prevalent and incident cohorts accumulated person-years of follow-up starting from the index date until 365 days after the index date. Patients who did not reach at least one year of follow-up were excluded from the study.

### Clinical indicators

Thirty-six quality indicators were developed and grouped in three categories of Accessibility and Appropriateness (n = 28), Continuity (n = 4) and Safety (n = 4) describing aspects of mental health care for people with bipolar disorders. The set of indicators were jointly developed by two multidisciplinary expert groups funded by the Italian Ministry of Health [19, 20]. The indicators were identified from the recommendations tailored to community care produced in agreement between the Ministry of Health and the Italian Regional Governments [23], considering the guidelines developed by the Canadian Psychiatric Association [24] and the National Institute for Clinical Excellence [25]. Thirty-two were process indicators identifiable from HCU registers, and the outcome indicators were mortality and those related to (re-)admission in GHPWs.

### Statistical analysis

Raw and age-gender standardized prevalence and incidence rates, and clinical indicators were computed for each region/province and for the aggregated sample. As calculations were performed separately for each area, summarized estimates were obtained by pooling data through meta-analytic procedures.

Both process and outcome indicators were measured and expressed as percentages (of patients to whom a specific healthcare intervention was provided) or medians (of interventions delivered), respectively assessing the proportion of patients who had access to a specific intervention and the intensity of delivery of that intervention.

The hypothesis of homogeneity across regional estimates of indicators was tested by means of chi-square test for indicators expressed both as proportions and as median of interventions for person-year of follow-up [26]. Heterogeneity of estimates across areas was assessed with the I^2^ statistics [27].

To evaluate continuity of pharmacological treatment, all prescriptions of mood stabilizers (see Supplementary Table S2 for ATC codes) dispensed during the follow-up period and reimbursed from the NHS were identified. The duration of each prescription was computed by dividing the total amount of prescribed drugs by the defined daily dose. We considered prescriptions as continuous if the interval between the end of one prescription and the start of the next one was shorter than 90 days, and interrupted if the time interval was longer than 90 days. Interrupted prescriptions were considered to lead to discontinuation of treatment.

To evaluate the continuity of community care, all contacts provided by services were identified. A patient was considered to receive continuous care if he/she had experienced at least one community contact every 90 days in the 365 days after the first contact in the follow-up. The time spent in hospital wards and residential facilities contributed to the period of continuity of care.

To assess mortality, the expected number of deaths was calculated by grouping female and male individuals by age (in 5-year age-interval groups) and multiplying the number of patients in each group by the corresponding age- and gender-specific mortality rates reported among the general population of each area in the year 2015 [http://demo.istat.it/index.html]. The standardized mortality ratio (SMR), which gives the ratio between observed and expected deaths, was then calculated. The corresponding 95% confidence intervals were computed on the assumption that the observed number of deaths followed a Poisson distribution.

Finally, indicators were also calculated according to gender for each area and the whole sample, and all the differences between men and women were tested by calculating the standardized mean difference (SMD), an alternative to p-value not influenced by the sample size. Standardized mean differences < 0.10 were considered as negligible and not statistically significant.

The SAS Software (version 9.4; SAS Institute, Cary, NC, USA), the Excel Software (from the Microsoft Office Personal Productivity Software Suite, Version 2019 16.0.6742.2048) and the R software (version 4.1.3/2022, R Foundation for Statistical Computing, Vienna, Austria; package: “*metamedian*”) were used to perform the analyses.

## Results

**Fig. 1:**
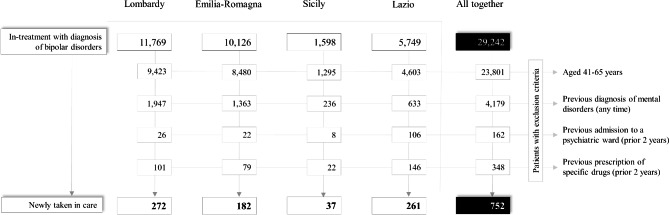
Flow-chart of inclusion and exclusion criteria for the eligibility of patients newly taken-in-care with bipolar disorder in three regions (Lombardy, Emilia-Romagna, Lazio) and one province (Palermo), and in the whole Italian sample. QUADIM-MAP Projects, Italy, 2015–2016

Figure 1 presents the flow from prevalent to incident cases. Prevalent cases were 29,242 and the incident ones were 752. Compared to prevalent, incident cases comprised more men (Table 1).

**Table 1:** Baseline characteristics of treated prevalent and incident cases with bipolar disorder in the whole sample. QUADIM-MAP projects, Italy, 2015–2016

	Prevalent cases (N = 29,242)	Incident cases (N = 752)
**Gender**		
Males	12,355 (42.3%)	384 (51.1%)
**Age (years)**		
Mean (SD)	53.4 (14.3)	23.76 (4.69)
18–25	1,433 (4.9%)	337 (44.8%)
26–40	4,995 (17.1%)	415 (55.2%)
41–50	6,672 (22.8%)	-
51–60	7,136 (24.4%)	-
> 60	9,006 (30.8%)	-
**Job condition**		
Working	12,229 (41.8%)	287 (38.2%)
Not working	11,336 (38.8%)	313 (41.6%)
Disability pension	2,822 (9.7%)	49 (6.5%)
*Missing data*	*2,855 (9.7%)*	*103 (13.7%)*
**Living arrangement** ^**§**^		
With family	17,022 (72.5%)	358 (72.9%)
Residential facility	752 (3.2%)	12 (2.4%)
Alone	3,474 (14.8%)	48 (9.8%)
*Missing data*	*2,245 (9.5%)*	*73 (14.9%)*
**Marital status**		
Never married	10,302 (35.2%)	501 (66.6%)
Married	12,218 (41.8%)	143 (19.0%)
Separated	1,953 (6.7%)	23 (3.1%)
Divorced	1,868 (6.4%)	7 (0.9%)
Widow/er	1,420 (4.8%)	2 (0.3%)
*Missing data*	*1,481 (5.1%)*	*76 (10.1%)*

^§^ Information from Lazio was not available for this variable, and percentages were computed on the remaining patients, i.e., respectively 23,493 and 491 for the treated prevalent and the incident cohort

### Accessibility and appropriateness

As shown in Table 2, the age- and gender-standardized treated prevalence rate was 14.6/10,000 individuals aged 18–65, and the incidence rate 1.4/10,000 individuals aged 18–40. Almost all prevalent cases had at least one outpatient/day care contact (96.7%) in the index year. The median of outpatient/day care contacts was 9.3 per person-year. Psychiatric visits were offered to 87.8% of the whole sample, at least 50% of patients had 4.8 psychiatric visits in the index year, and 10.4% of prevalent patients received at least one home visit. Whereas some psychosocial interventions were provided to slightly less than half of the prevalent cases, psychoeducation was provided to 3.5% and psychotherapy to 11.5%. For 30.6% of patients there was at least one contact with a family member. Median values of psychoeducation and psychotherapy sessions were 2.2 and 4.8 respectively and 2.0 for contacts with the family members. These values were similar to those found in incident cases (Table 3).

**Table 2:** Estimated values of indicators for treated prevalent patients with bipolar disorder in 2015 in four Italian areas (Lombardy, Emilia-Romagna, Lazio and Province of Palermo) and in the whole sample. QUADIM-MAP projects, Italy, 2015–2016

		Lombardy (n = 11,769)	Emilia-Romagna (n = 10,126)	Lazio (n = 5,749)	Palermo (n = 1,598)	Whole sample (n = 29,242)	I^2^ ¥
	Crude treated prevalence	14.2	27.1	11.7	15.3	16.2	
	Age and gender standardized one-year treated prevalence rate (x 10,000)	12.8	21.8	12.3	13.3	14.6	
**accessibility and appropriateness**							
*1*	Patients with at least one outpatient contact	96.9%	98.9%	97.5%	78.3%	96.7%	99
*2*	Median number of outpatient contacts	7.0	13.0	10.0	7.0	9.3	99
*3*	Patients receiving at least one psychiatric visit	91.5%	85.1%	89.0%	73.9%	87.8%	99
*4*	Median number of psychiatric visits	5.0	4.0	6.0	4.0	4.8	99
*5*	Patients with at least one home visit **§**	10.2%	-	6.8%	11.7%	10.4%	93
*6*	Median number of home visits **§**	4.0	-	2.0	2.0	2.7	99
*7*	Patients receiving any psychosocial interventions	45.4%	47.7%	47.7%	54.1%	47.1%	94
*8*	Median number of psychosocial interventions	4.0	3.0	3.0	3.0	3.3	97
*9*	Patients receiving psychoeducation **‡**	2.9%	3.8%	NA	5.6%	3.5%	89
*10*	Median number of psychoeducation sessions **‡**	2.5	2.0	NA	2.0	2.2	98
*11*	Patients receiving psychotherapy	14.3%	5.5%	16.5%	10.5%	11.5%	99
*12*	Median number of psychotherapy sessions	6.0	4.0	6.0	3.0	4.8	99
*13*	Patients whose relatives had at least one contact	30.7%	33.2%	20.5%	48.7%	30.6%	99
*14*	Median number of contacts with relatives	2.0	2.0	2.0	2.0	2.0	0
*15*	Patients treated with antipsychotics	62.4%	67.8%	58.3%	57.4%	63.2%	98
*16*	FGAs	11.2%	18.8%	13.0%	11.7%	14.2%	99
*17*	SGAs	58.2%	59.7%	55.2%	53.2%	57.8%	93
*18*	Patients treated with mood stabilizers	75.0%	73.3%	63.3%	68.3%	71.5%	99
*19*	Lithium	26.7%	20.7%	14.3%	14.4%	21.5%	99
*20*	Other mood stabilizers	65.4%	67.0%	59.7%	64.8%	64.8%	97
*21*	Patients treated with antidepressants	45.0%	57.7%	32.2%	39.4%	46.6%	99
*22*	Patients treated with mood stabilizers without any other intervention	1.4%	0.8%	1.6%	10.8%	1.7%	98
*23*	Patients with at least one admission in residential facilities	15.4%	13.1%	11.6%	0.9%	13.1%	99
*24*	Median number of days in residential facilities	29.0	35.0	46.0	145.1	63.5	99
*25*	Patients with at least one admission in GHPW	13.1%	10.1%	11.8%	12.5%	11.8%	94
*26*	Median number of days in GHPW	34.8	28.6	74.0	21.4	17.2	97
*27*	Admissions with a length of stay in GHPW higher than 30 days	9%	5%	7%	3%	7%	92
*28*	Unplanned re-admissions in GHPW within 30 days^**¶**^	20%	18%	23%	18%	20%	59
**Continuity of care**							
*29*	Patients with continuous community care	57.7%	60.0%	53.8%	33.5%	60.7%	99
*30*	Patients persistent with mood stabilizers therapy	48.0%	67.7%	59.5%	71.2%	58.2%	99
*31*	GHPW discharges followed by an outpatient contact within 14 days	60%	76%	61%	43%	62%	98
*32*	GHPW discharges followed by home care within 14 days **§**	5%	NA	6%	2%	5%	82
**SAFETY**							
*33*	Patients assessed for hyperglycaemia and hyperlipidaemia *(in patients treated with antipsychotics)*	28.0%	32.5%	23.5%	30.8%	29.0%	97
*34*	Patients assessed for lithium level *(in patients treated with lithium)*	78.9%	71.6%	51.5%	60.9%	72.2%	99
*35*	Mortality (Number of deaths)	140 (1.2%)	148 (1.5%)	79 (1.4%)	14 (0.9%)	381 (1.3%)	54
*36*	Mortality (SMR, and relative 95% CI)	1.37 (1.22 to 1.53)	1.26 (1.13 to 1.40)	1.59 (1.36 to 1.84)	1.08 (0.73 to 1.55)	1.35 (1.26 to 1.44)	

DMH: Department of Mental Health; CMHC: Community Mental Health Centres; DC: Day-Care Centres; PY: person-year; FGAs: first generation antipsychotics; SGAs: second generation antipsychotics; GHPW: General Hospital Psychiatric Wards; SMR: standardized mortality ratio

* P-value < 0.05 for test of homogeneity across areas

**§** Information for Emilia-Romagna was not available for this indicator, and percentages were computed on the 19,116 remaining patients

**‡** Information for Lazio was not available for this indicator, and percentages were computed on the 23,493 remaining patients

**¶** After a previous hospital admission in GHPW (statistical unit)

**¥** Values of I^2^ for heterogeneity can be classified in negligible (0–25); moderate (26–50); substantial (51–75); considerable (76–100)

**Table 3:** Estimated values of indicators for incident patients with bipolar disorder in four Italian areas (Lombardy, Emilia Romagna and Lazio Regions and Province of Palermo) and in the whole sample. QUADIM-MAP projects, Italy, 2015–2016

		Lombardy (n = 272)	Emilia-Romagna (n = 182)	Lazio (n = 261)	Palermo (n = 37)	Whole sample (n = 752)	I^2^ ¥
	Crude treated incidence	1.1	1.7	1.7	1.0	1.3	
	Age and gender standardized one-year treated incidence rate (x 10,000)	0.8	1.5	1.9	1.2	1.4	
**ACCESSIBILITY AND APPROPRIATENESS**							
*1*	Patients with at least one outpatient contact	84.2%	97.8%	97.5%	89.2%	91.9%	93
*2*	Median number of outpatient contacts	10.0	11.0	8.0	8.0	8.6	0
*3*	Patients receiving at least one psychiatric visit	79.0%	93.4%	88.1%	86.5%	86.0%	87
*4*	Median number of psychiatric visits	6.0	6.0	6.0	3.5	5.5	74
*5*	Patients with at least one home visit **§**	1.8	-	13.5%	6.9%	4.9%	99
*6*	Median number of home visits **§**	2.0	-	2.0	1.0	1.7	95
*7*	Patients receiving any psychosocial interventions	45.2%	41.2%	47.9%	75.7%	46.7%	85
*8*	Median number of psychosocial interventions	6.0	4.0	3.0	2.0	3.8	86
*9*	Patients receiving psychoeducation **‡**	4.8%	5.5	-	8.1%	5.3%	22
*10*	Median number of psychoeducation sessions **‡**	2.0	2.5	-	2.0	2.2	57
*11*	Patients receiving psychotherapy	25.4%	11.5%	19.5%	21.6%	19.8%	95
*12*	Median number of psychotherapy sessions	5.0	7.7	4.0	5.5	5.5	78
*13*	Patients whose relatives had at least one contact	32.0%	29.1%	26.8%	73.0%	31.5%	92
*14*	Median number of contacts with relatives	3.0	4.0	2.0	2.0	2.7	92
*15*	Patients treated with antipsychotics	52.4%	62.6%	42.1%	40.5%	50.7%	86
*16*	FGAs	4.8%	17.6%	8.8%	2.7%	9.2%	86
*17*	SGAs	51.5%	56.0%	39.8%	40.5%	48.0%	79
*18*	Patients treated with mood stabilizers	62.9%	73.6%	43.3%	51.4%	58.1%	94
*19*	Lithium	23.2%	17.6%	9.2%	0.0%	15.8%	91
*20*	Other mood stabilizers	58.5%	67.0%	41.4%	51.4%	54.3%	91
*21*	Patients treated with antidepressants	35.3%	41.8%	21.1%	27.0%	31.5%	89
*22*	Patients treated with mood stabilizers without any other intervention	4.0%	1.6%	1.1%	0.0%	2.3%	81
*23*	Patients with at least one admission in residential facilities	18.4%	18.7%	16.9%	0.0%	17.0%	98
*24*	Median number of days in residential facilities	16.0	32.0	34.5	0.0	27.0	92
*25*	Patients with at least one admission in GHPW	18.4%	20.9%	13.0%	10.8%	16.8%	55
*26*	Median number of days in GHPW	14.0	25.0	14.0	13.0	16.5	96
*27*	Admissions with a length of stay in GHPW higher than 30 days	1.6%	9.4%	0%	0%	3.0%	30
*28*	Unplanned re-admissions in GHPW within 30 days^**¶**^	9.5%	15.6%	35.3%	0%	17.4%	90
**CONTINUITY OF CARE**							
*29*	Patients with continuous community care	51.1%	30.3%	34.3%	24.2%	38.4%	99
*30*	Patients persistent with mood stabilizers therapy	36.3%	45.5%	35.4%	52.6%	39.6%	18
*31*	GHPW discharges followed by an outpatient contact within 14 days	60.3%	71.9%	52.9%	33.3%	60.6%	32
*32*	GHPW discharges followed by home care within 14 days **§**	0	-	2.9%	0%	1.0%	38
**SAFETY**							
*33*	Patients assessed for hyperglycaemia and hyperlipidaemia *(in patients treated with antipsychotics)*	9.2%	13.2%	10.0%	0.0%	10.2%	93
*34*	Patients assessed for lithium level *(in patients treated with lithium)*	77.8%	75.0%	37.5%	0.0%	68.9%	85
*35*	Mortality (Number of deaths)	0 (0%)	1 (0.5%)	0 (0%)	1 (0.4%)	2 (0.3%)	0

DMH: Department of Mental Health; CMHC: Community Mental Health Centres; DC: Day-Care Centres; PY: person-year; FGAs: first generation antipsychotics; SGAs: second generation antipsychotics; GHPW: General Hospital Psychiatric Wards; SMR: standardized mortality ratio

* P-value <0.05 for test of homogeneity across areas

**§** Information for Emilia-Romagna was not available for this indicator, and percentages were computed on the 570 remaining patients

**‡** Information for Lazio was not available for this indicator, and percentages were computed on the 491 remaining patients

**¶** After a previous hospital admission in GHPW (statistical unit)

**¥** Values of I^2^ for heterogeneity can be classified in negligible (0–25); moderate (26–50); substantial (51–75); considerable (76–100)

63% of prevalent cases were treated with antipsychotics, 71.5% with mood stabilizers (with lithium prescribed to about one fifth of the sample), and 46.6% with antidepressants (Table 2). Incident cases who received at least one prescription were slightly less than prevalent cases. Eventually all prevalent and incident cases who were prescribed with a mood stabilizer had at least one contact in the period, with differences across areas.

Admissions to residential facilities concerned 13.1% of prevalent cases (Table 2) with a median stay of 63.5 days. In incident cases, 17% had at least one admission and lengths of stay were shorter than in prevalent cases (Table 2). 11.8% of prevalent cases were admitted to GHPWs at least once in the index period. Admissions to GHPW were slightly more frequent in incident cases (16.8%) but length of stay was shorter.

For almost all indicators heterogeneity across areas was substantial or considerable, either in prevalent and incident cases (Tables 2 and 3).

### Continuity

Care was offered continuously to 60.7 of prevalent cases. Continuity of prescription of mood stabilizers was reported for 58% of prevalent cases, and 39.6% of incident cases. 62% of admissions of prevalent cases to GHPW were followed by an outpatient/day care contact within 14 days after discharge, and 5% by a home visit (Table 2). The corresponding values in incident cases were 60.6% and 1% (Table 3). Heterogeneity across areas was considerable for all indicators in both cohorts.

### Safety

Among patients who were prescribed with antipsychotics, 29% were assessed at least once in the year for hyperglycemia and hyperlipidemia, and 72.2% of those prescribed with lithium for lithium level. One in ten incident cases with an antipsychotic drug therapy was monitored for hyperglycaemia and hyperlipidemia. Heterogeneity across areas was considerable in both cohorts.

In the follow-up there were 381 deaths among prevalent cases (1.3%), corresponding to a SMR of 1.35, ranging from 1.08 (95% CI: 0.73–1.55) in the province of Palermo to 1.37 (95% CI: 1.22–1.53) in Lombardy (Table 2).

### Differences by gender

Treated prevalence rate in men was 14.3/10,000 individuals, 18.0 in women. Differences by gender in prevalent cases were slight and not statistically significant in most indicators (Supplementary Table S3). Even where statistical significance was present, the differences were small, with the only large difference in use of antidepressants (40% in males and 51.3% in females). In prevalent cases, males showed a significant excess in mortality rate of 60% (95% CI: 45-77%) compared to the general population, whereas for females the excess of mortality was 18% (95% CI: 7-29%).

## Discussion

We found a one-year treated prevalence of 0.15% for bipolar disorders in 2015, higher than the rate of 0.12% reported by the Ministry of Health for the entire country in the same year [28]. The few available data show higher treatment rates, ranging from 0.36% in males to 0.58% in females in Sweden [29], and being 0.45% in Taiwan [30] and 0.53% in Canada [8]. The peculiar difficulties in the epidemiology of bipolar disorders - high frequency of misdiagnosis [9], frequent use of office-based private care, the up-and-down nature of the disorder - can explain this difference. The one-year true prevalence of bipolar disorders in Europe was estimated between 0.8% [31] and 1.7% [6, 32]. Assuming these estimates for Italy, a treatment gap of 80–90% would result. The findings of this study add to the consolidated observation of a meaningful treatment gap in bipolar disorders, which is higher than the gap of 65% estimated for Germany [33]. Treated incidence rate was 1.4/10,000, lower than the 2.8/10,000 rate reported by the Ministry of Health for 2015 [28], and of 5.2/10,000 in females and 3.2/10,000 in males in a Swedish registry [29].

The great majority of patients had at least one outpatient or day care contact in the year, suggesting that some coverage by community services on a whole was assured, with more than half of the patients having nine contacts in the year. However, continuity of care was low, with 40% of patients visited less than once every three months.

About half of people received some psychosocial interventions, including different activities. Psychoeducation and psychotherapy were offered to few patients, particularly if compared to other studies, where psychoeducation was offered to about 17% of prevalent patients with bipolar disorders in a year [34]. The median of 2.2 sessions of psychoeducation in both cohorts shows a low frequency, suggesting that real-world practice may be far from indications derived from to the models tested in clinical trials [35], consisting of 21 sessions. Psychoeducation delivery seems to be at odds also with the Italian recommendations [23] which indicates to provide psychoeducation and support on a routine basis to all patients with bipolar disorders. The medians of 4.8 psychotherapy sessions in prevalent cases and 5.5 in incident cases were lower than the number of sessions considered minimally adequate [36, 37]. Contacts with the family were infrequent in both samples, and particularly worrying in incident cases, where 73% were living with their family. A previous paper focused on Lombardy in 2009 found that 8% of patients with bipolar disorders had at least one session of psychotherapy [11]. However, it is possible that some prevalent cases not receiving psychotherapy or psychoeducation in the index year had received it before, since both interventions are not delivered continuously.

The study uses data related to 2015, when the guidelines of reference were those issued before that year. CANMAT 2013 guidelines. Indeed, some differences do exist between the CANMAT guidelines of 2013 and the 2018, where for psychological interventions in the maintenance phase of bipolar disorders psychoeducation is recommended as first line, cognitive behavioural therapy and family focused as second line [38]. Unfortunately, psychotherapy model is not indicated in our database.

Italian recommendations indicate to offer psychotherapy “to whom might benefit” from it, without mentioning patients’ preferences. In spite of the professional’s discretion supported by the Italian guidelines, the figures of 11.5% in prevalent and 19.8% in incident cases seem low and may suggest professionals’ little attention to this intervention and a poor availability of the necessary resources.

There are uncertainties in research in drug treatment of bipolar disorder [12]. Guidelines recommend a variety of drugs in monotherapy or in combination according to the different phases and features of the disorder. In our study, the lack of information on clinical indication for prescriptions limited the possibility of a thorough quality assessment. Second-generation antipsychotics, lithium and so-called mood stabilizers all play a role throughout the phases of illness. However, lithium is considered the gold standard, especially for long-term treatment [24, 25]. The use of antidepressants remains controversial, due to limited efficacy and risk to switch into hypomania or full-blown mania [10].

Lithium was prescribed to a minority of patients, but the comparison with a past survey in Lombardy shows that it is not declining [39] suggesting that concerns for the decreasing use of lithium is not confirmed by these data [40]. However, the rate of lithium prescriptions remained low.

In our sample, the most frequently prescribed drugs were mood stabilizers, followed by second-generation antipsychotics. The pattern was the same in incident cases but with fewer prescribed patients, suggesting that professionals may have a wait-and-see attitude or offer more frequently alternative options to young people, like psychosocial interventions.

The drug utilization patterns emerging from this study highlight three main areas for quality improvement: the low continuity of drug treatment; the high rate of antidepressants prescriptions in both samples; the lack of attention to adverse effects shown by low rates of metabolic risks assessment. Fewer incident than prevalent cases received continuous care. Such a difficulty in maintaining continuity might be related to a less assertive approach with young patients or to a more difficult engagement. Differences in pharmacological treatment in males and females were remarkable for antidepressants, which were prescribed to 51% of women versus 40% in men. These findings are in agreement with a study comparing males and females in a Swedish sample [41], which suggested that the wider use of antidepressants in women was related to the higher frequency of depression.

Mortality in the group of prevalent cases was 35% higher than in the general population, in agreement with other reports [42, 43]. Differences in mortality between males and females were considerable, with a 60% excess mortality in men and 18% in females compared to the general population. Females showed slightly more favorable indicators relative to care, continuity of care and safety, and this may have an impact on physical health. Unfortunately, we do not have information about causes of death, therefore we cannot speculate about the causes of gender differences in mortality risk. It would be possible that higher risk for cardiovascular diseases and, to a lesser extent, higher mortality by suicides in men (39, 40) are both contributing to this difference.

The differences observed among areas can be at least partly due to differences in resources of the DMHs. According to national data [23, 28] the rate of personnel per 100,000 inhabitants was highest in Emilia-Romagna (86.5), where we found the most favorable indicators, 60.6 in Lombardy, 45 in Lazio, and 70.5 in Sicily (that we can consider as representative of the province of Palermo). The difference was wide in number of educators between Emilia-Romagna (8.7/100,000) and Sicily (1.1).

Our study is based on a large, unselected cohort from an area covering about one-fifth of the national population. It is the largest evaluation carried out in Italy on the quality of mental health care delivered to patients with bipolar disorders, and it is among the most extensive surveys conducted in Western countries [8, 44]. We could assess the complete care pathway of patients with bipolar disorders thanks to the availability of high-quality integrated individual data. Treated incident patients were identified from their first contact with the NHS mental health services where a bipolar disorder was diagnosed. Nonetheless, we were able to detect only the first contact registered in public mental health services, maybe missing diagnoses made in private settings or in areas not covered by our investigation. Other limitations concern the lack of clinical data and lack of information about treatments provided by private organizations, and non-reimbursable payments. Indeed, common sources of exposure misclassification when using HCU data include drug treatments dispensed by private services, as well as out-of-pocket payments and over the counter (OTC) medications. However, OTC drug purchase in Italy is not allowed for mood stabilizers, antidepressants and antipsychotics; despite that, by including all the prescriptions related to these drugs reimbursed from the NHS we have been able to track the overwhelming majority of drug prescriptions. It should be also emphasized that, since HCU data are used to reimburse accredited and public service providers, incorrect and incomplete reporting leads to legal consequences. Some between-region differences may be partly due to heterogeneity in the quality and completeness of data [19]. When the study and the data management of the HCU used started the most recent databases available were related to the years 2013–2016. Some differences may have occurred since 2015, but not of such an extent to materially modify or invalidate our findings.

These findings enable a wide pictures of mental health care offered to people with bipolar disorders. Indicators of strategies clearly oriented to autonomy and social inclusion are not provided, therefore indicating areas of information needing improvement. We know very little about shared care and involvement of users in service evaluation, inclusion of experts by experience in some activities, about intensity and quality of professionals’ training [45]. Patients’ outcomes are also missing in these databases.

## Conclusions

These data confirm a treatment gap in bipolar disorders in Italian mental health services, higher than in other countries. This gap occurs in a mental health system of care entirely community-based, suggesting that this does not assure sufficient coverage by itself. For those who are in care, continuity of contact looks sufficient, but intensity of care is low and poor adherence to evidence-based guidelines is shown by low rate of access to psychotherapies and lithium treatment, suggesting suboptimal care and risk of limited effectiveness. We also confirmed that women have higher prevalence rates but lower mortality than men.

Large databases allow monitoring the activities of mental health services, in ordinary and in exceptional times. For instance, they will be extremely useful in describing the expected decrease in activity during the COVID-19 pandemic and how long the services will take to recover the pre-COVID intensity of care. Given the widespread implementation of services for early intervention in severe disorders, it would be useful to assess whether access to care of young people has really been facilitated. Real-world data also allow benchmarking, that should not be just a sort of academic exercise, rather prompt comparison and understanding of specific aspects in order to remove problems and reach higher standards [16, 46].

### Electronic supplementary material

Below is the link to the electronic supplementary material.

Supplementary Material 1. **Table S1**: Service interventions, treatments and activities delivered by Community Mental Health Centers (CMHCs) and Day Centers (DCs), and their classification in the Italian Mental Health Information System; **Table S2**: Diagnostic and therapeutic (ICD-9-CM, ICD-10, and ATC) codes used in the current study for drawing records and fields from Healthcare Utilization databases; **Table S3**: Estimated values of clinical indicators for treated prevalent patients with bipolar disorder according to gender (and in the whole sample). QUADIM-MAP projects, Italy, 2015-2016.

## Data Availability

The data that support the findings of this study are available from the Regions of Lombardy, Lazio and Emilia-Romagna, and the Province of Palermo, but restrictions apply to the availability of these data, which were used under license for the current study, and so are not publicly available. Data are however available from the authors upon reasonable request and with permission of the Regions involved in this study. The reference contacts for this issue are the Corresponding Author (MMC) and some co-authors (GC, giovanni.corrao@unimib.it; AL, a.lora@asst-lecco.it; AS, alessio.saponaro@regione.emilia-romagna.it; AG, andrea.gaddini@gmail.com; SS, salvatore.scondotto@gmail.com) who abstracted the data from the Regions involved and authorized their use.

## Abbreviations

ANOVA: Analysis of Variance
ATC: Anatomical Therapeutic Chemical Classification System
CMHCs: Community Mental Health Centers
CRFs: Community Residential Facilities
DCs: Day-Care Centers
DMHs: Departments of Mental Health
GHPWs: General Hospital Psychiatric Wards
HCU: Healthcare Utilization databases
ICD: International Classification of Diseases
MHIS: Mental Health Information System
MoH: Ministry of Health
NHS: National Health Service
QUADIM: “Quality of clinical pathways in patients with severe mental disorders in Italy” project
SAS: Statistical Analysis System software
SMR: Standardized Mortality Ratio
