# Supplementary material for: The quality of mental health care for people with bipolar disorders in the Italian mental health system: the QUADIM project
Source: BMC Psychiatry. 2023 Jun 13;23:424. doi: 10.1186/s12888-023-04921-7 (PMC10261835; doi:10.1186/s12888-023-04921-7)
Supplement: Supplementary file 1 — Supplementary Material 1. Table S1: Service interventions, treatments and activities delivered by Community Mental Health Centers (CMHCs) and Day Centers (DCs), and their classification in the Italian Mental Health Information System; Table S2: Diagnostic and therapeutic (ICD-9-CM, ICD-10, and ATC) codes used in the current study for drawing records and fields from Healthcare Utilization databases; Table S3: Estimated values of clinical indicators for treated prevalent patients with bipolar disorder according to gender (and in the whole sample). QUADIM-MAP projects, Italy, 2015-2016. [file 12888_2023_4921_MOESM1_ESM.docx]

**Supplementary Table S1.** Service interventions, treatments and activities delivered by Community Mental Health Centers (CMHCs) and Day Centers (DCs), and their classification in the Italian Mental Health Information System.

| **Interventions and activities** | **Mental Health Information system code** |
| --- | --- |
|  |  |
| **Generic care** |  |
| Psychiatric visit | 01 |
| Individual meeting with a professional | 03 |
| Consultation | 04 |
| Medico-legal assessment | 05 |
| Psychological testing | 06 |
| Drug administration | 11 |
| Meeting with relatives | 12 |
| Staff meeting | 15 |
| Attendance to day centre | 20 |
| Support to daily living activity | 24 |
| Network interventions | 26 |
| **Psychosocial interventions** |  |
| Individual living skills training | 16 |
| Group living skills training | 17 |
| Individual socialization | 18 |
| Socialization group | 19 |
| Expressive, manual and bodywork individual interventions | 21 |
| Expressive, manual and bodywork group interventions | 22 |
| Vocational training | 23 |
| Support for financial, welfare procedures and leisure activities | 25 |
| **Psychotherapy** |  |
| Psychological visit | 02 |
| Individual psychotherapy | 07 |
| Couple psychotherapy | 08 |
| Family psychotherapy | 09 |
| Group psychotherapy | 10 |
| **Psychoeducation** |  |
| Single family psychoeducation | 13 |
| Multifamily group psychoeducation | 14 |

**Supplementary Table S2.** Diagnostic and therapeutic (ICD-9-CM, ICD-10, and ATC) codes used in the current study for drawing records and fields from Healthcare Utilization databases.

| **DIAGNOSES** | |
| --- | --- |
|  | **ICD-10 codes**  **(Lombardy)** |
| Manic episode | F30.* |
| Bipolar affective disorder | F31.* |
| Cyclothymia | F34.0 |
| Other single mood [affective] disorders | F38.0 |
|  | **ICD-9-CM codes**  **(Emilia-Romagna, Lazio and Palermo)** |
| Bipolar I disorder, single manic episode | 296.0 |
| Manic disorder, recurrent episode | 296.1 |
| Bipolar I disorder, most recent episode (or current) manic | 296.4 |
| Bipolar I disorder, most recent episode (or current) depressed | 296.5 |
| Bipolar I disorder, most recent episode (or current) mixed | 296.6 |
| Bipolar I disorder, most recent episode (or current) unspecified | 296.7 |
| Bipolar disorder, unspecified | 296.80 |
| Atypical manic disorder | 296.81 |
| Other | 296.89 |
| Other specified episodic mood disorder | 296.99 |
| Excitative type psychosis | 298.1 |
| **DRUGS** | |
|  | **ATC codes** |
|  |  |
| Mood Stabilizers | N05AN, N03AX09, N03AG01, N03AF01, N05AH03, N05AH04, N05AX12 |
| Lithium | N05AN |
| Lamotrigine | N03AX09 |
| Valproic acid, carbamazepine | N03AG01, N03AF01 |
| First generation antipsychotics (FGAs) | N05AA01, N05AA02, N05AA03, N05AA04, N05AA05, N05AA06, N05AA07, N05AB01, N05AB02, N05AB03, N05AB04, N05AB05, N05AB06, N05AB07, N05AB08, N05AB09, N05AB10, N05AC01, N05AC02, N05AC03, N05AC04, N05AD01, N05AD02, N05AD03, N05AD04, N05AD05, N05AD06, N05AD07, N05AD08, N05AD09, N05AE01, N05AE02, N05AE03, N05AF01, N05AF02, N05AF03, N05AF04, N05AF05, N05AL01, N05AL02, N05AL03, N05AL04, N05AL05, N05AL06, N05AL07 |
| Second generation antipsychotics (SGAs) |  |
| Olanzapine | N05AH03 |
| Quetiapine | N05AH04 |
| Aripiprazole | N05AX12 |
| Others | N05AE04, N05AE05, N05AH01, N05AH02, N05AH05, N05AX07, N05AX08, N05AX10, N05AX11, N05AX13, N05AX14, N05AX15 |
| **LABORATORY TESTS** | |
|  | **National procedure codes** |
| Glycated haemoglobin | 90.27.1, 90.28.1 |
| Lipid profile | 90.14.1, 90.14.3, 90.43.2 |
| Electrolytes | 91.49.2, 90.40.4, 90.37.4, 90.13.3 |
| Lithaemia | 90.32.2 |
| Complete blood count | 90.62.2 |
| Liver function assessment | 90.04.5, 90.09.2, 90.25.5 |

**Supplementary Table S3.** Estimated values of clinical indicators for treated prevalent patients with bipolar disorder according to gender (and in the whole sample). QUADIM-MAP projects, Italy, 2015-2016.

|  |  | **Males**  (n=12,355) | **Females**  (n=16,887) | **SMD** |
| --- | --- | --- | --- | --- |
|  | Age-standardized treated prevalence | 14.3 | 18.0 |  |
| **ACCESSIBILITY AND APPROPRIATENESS** | |  |  |  |
| *1* | Patients with at least one outpatient contact | 96.6% | 96.8% | 2.0 |
| *2* | Median number of outpatient contacts | 9.3 | 9.3 | 0.0 |
| *3* | Patients receiving at least one psychiatric visit | 88.1% | 87.6% | 2.5 |
| *4* | Median number of outpatient psychiatric visits | 4.8 | 4.8 | 0.0 |
| *5* | Patients with at least one home visit **§** | 9.2% | 11.3% | 9.3 |
| *6* | Median number of home visit **§** | 2.7 | 3.3 | 0.0 |
| *7* | Patients receiving any psychosocial intervention | 47.5% | 46.9% | 1.8 |
| *8* | Median number of psychosocial interventions | 3.3 | 3.5 | 0.0 |
| *9* | Patients receiving psychoeducation **‡** | 3.8% | 3.2% | 4.7 |
| *10* | Median number of psychoeducation sessions**‡** | 2.5 | 2.0 | 0.0 |
| *11* | Patients receiving psychotherapy | 10.8% | 11.9% | 4.9 |
| *12* | Median number of psychotherapy sessions | 4.0 | 5.3 | 0.1 |
| *13* | Patients whose relatives had at least one contact | 30.7% | 30.5% | 0.6 |
| *14* | Median number of contacts with relatives | 2.2 | 1.7 | 0.1 |
| *15* | Patients treated with antipsychotics | 63.8% | 62.7% | 3.3 |
| *16* | FGAs | 14.5% | 14.0% | 2.3 |
| *17* | SGAs | 58.6% | 57.3% | 3.6 |
| *18* | Patients treated with mood stabilizers | 72.3% | 71.0% | 3.8 |
| *19* | Lithium | 23.0% | 20.4% | 9.3 |
| *20* | Other mood stabilizers | 64.8% | 64.8% | 0.2 |
| *21* | Patients treated with antidepressants | 40.0% | 51.3% | 32.0***** |
| *22* | Patients treated only with mood stabilizers without any other intervention | 1.8% | 1.6% | 1.7 |
| *23* | Patients with at least one admission to residential facilities | 14.0% | 12.4% | 6.6 |
| *24* | Median number of days in residential facilities | 65.6 | 82.0 | 0.1 |
| *25* | Patients with at least one admission to GHPW | 12.5% | 11.2% | 6.2 |
| *26* | Median number of days in GHPW | 17.2 | 17.8 | 0.0 |
| *27* | Admissions to GHPW longer than 30 days | 6.3% | 8.4% | 10.8***** |
| *28* | Unplanned re-admissions in GHPW within 30 days**^¶^** | 22.4% | 18.0% | 16.2***** |
| **CONTINUITY** | |  |  |  |
| *29* | Patients with continuous community care | 61.5% | 60.1% | 4.1 |
| *30* | Patients persistent with mood stabilizers therapy | 56.9% | 59.1% | 6.4 |
| *31* | GHPW discharges followed by an outpatient contact within 14 days | 62.0% | 62.8% | 2.2 |
| *32* | GHPW discharges followed by home care within 14 days **§** | 4.4% | 5.6% | 7.7 |
| **SAFETY** | |  |  |  |
| *33* | Patients assessed for hyperglycaemia and hyperlipidaemia  *(in patients treated with antipsychotics)* | 28.0% | 29.8% | 5.6 |
| *34* | Patients assessed for lithium level  *(in patients treated with lithium)* | 70.6% | 73.6% | 9.9 |
| *35* | Mortality (number of deaths) | 184 (1.5%) | 197 (1.2%) | 4.3 |
| *36* | Mortality (SMR, and relative 95% CI) | 1.60 (1.45 to 1.77) | 1.18 (1.07 to 1.29) | - |
|  |  |  |  |  |

SMD: Standardized Mean Difference; DMH: Department of Mental Health; CMHC: Community Mental Health Centres; DC: Day-Care Centres; PY: person-year; FGAs: first generation antipsychotics; SGAs: second generation antipsychotics; GHPW: General Hospital Psychiatric Wards; SMR: standardized mortality ratio.

***** Standardized mean differences ≥10% are considered not negligible for differences between males and females distributions.

**§** Information for Emilia-Romagna Region was not available for this indicator, and percentages were computed on the 19,116 remaining patients.

**‡** Information for Lazio Region was not available for this indicator, and percentages were computed on the 23,493 remaining patients.

**¶** After a previous hospital admission in GHPW (statistical unit).
